# The Single-molecule long-read sequencing of *Scylla paramamosain*

**DOI:** 10.1038/s41598-019-48824-8

**Published:** 2019-08-27

**Authors:** Haifu Wan, Xiwei Jia, Pengfei Zou, Ziping Zhang, Yilei Wang

**Affiliations:** 1 Key Laboratory of Healthy Mariculture for the East China Sea, Ministry of Agriculture and Rural Affairs, Fisheries College, Jimei University, Xiamen, 361021 P.R. China; 2 College of Animal Science, Fujian Agriculture and Forestry University, Fuzhou, 350002 P.R. China

**Keywords:** RNA splicing, Long non-coding RNAs

## Abstract

*Scylla paramamosain* is an important aquaculture crab, which has great economical and nutritional value. To the best of our knowledge, few full-length crab transcriptomes are available. In this study, a library composed of 12 different tissues including gill, hepatopancreas, muscle, cerebral ganglion, eyestalk, thoracic ganglia, intestine, heart, testis, ovary, sperm reservoir, and hemocyte was constructed and sequenced using Pacific Biosciences single-molecule real-time (SMRT) long-read sequencing technology. A total of 284803 full-length non-chimeric reads were obtained, from which 79005 high-quality unique transcripts were obtained after error correction and sequence clustering and redundant. Additionally, a total of 52544 transcripts were annotated against protein database (NCBI nonredundant, Swiss-Prot, KOG, and KEGG database). A total of 23644 long non-coding RNAs (lncRNAs) and 131561 simple sequence repeats (SSRs) were identified. Meanwhile, the isoforms of many genes were also identified in this study. Our study provides a rich set of full-length cDNA sequences for *S*. *paramamosain*, which will greatly facilitate *S*. *paramamosain* research.

## Introduction

*Scylla paramamosain* is an important aquaculture crab and has great economical and nutritional value. According to the statistics result, it had been estimated that the aquaculture production of *S*. *paramamosain* reached approximately 157,712 tons in China in 2018 (China Fishery Statistical Yearbook 2019). Up to date, the genome information for most crustaceans is not available. However, the application of second-generation sequencing technologies that do not need genome data has greatly accelerated the research of the crustacean. In crab and shrimp, the high-throughput sequencing technology has been applied in *Eriocheir sinensis*^1–21^, *Portunus trituberculatus*^22–29^, *S*. *paramamosain*^30–33^, *S*. *olivacea*^34^, *Carcinus maenas*^35,36^, *Gecarcinus lateralis*^37–41^, *P*. *sanguinolentus*^42^, *Charybdis feriatus*^43^, *Litopenaeus vannamei*^44–48^, *Macrobrachium rosenbergii*^49–52^, *M*. *nipponense*^53,54^, *Exopalaemon carinicauda*^55–57^, *Oratosquilla oratoria*^58–60^, *Homarus americanus*^61^, and so on. Many genes related with reproduction, growth, and immunity of crab and shrimp have been obtained through the transcriptome data.

However, the length of sequencing reads obtained using the second-generation sequencing technologies was usually short (usually 100–250 bp), which needs further bioinformatics analysis to assemble using the software such as Trinity to obtain the transcript sequence^62^. But it had been estimated that many repetitive elements exist in the crustacean genome DNA^63,64^, which could influence the assembled result, such as the undesirable N50 length of assembled unigenes and the majority of non-full-length transcript sequences.

The third-generation sequencing technology is also called the single-molecule real-time sequencing technology which include smart sequencing and nanopore sequencing developed by Pacific Biosciences and Oxford Nanopore Technologies, respectively. Compared to the second-generation sequencing technologies, the third-generation sequencing technology has many advantages, such as (1) the longer sequencing length, (2) the obtainment of full-length transcripts, (3) the direct sequencing without the need for fragmentation or post-sequencing assembly, (4) the analysis of alternative splicing^65^. But up to date, the application of the third-generation sequencing technology in crustacean is scare.

In this study, a RNA library consisted of multiple tissues of *S*. *paramamosain* (gill, hepatopancreas, muscle, cerebral ganglion, eyestalk, thoracic ganglia, intestine, heart, testis, ovary, sperm reservoir and hemocyte) was constructed and sequenced using the third-generation sequencing technology (Pacbio) for the first time, which would not only further enrich the genetic information and promote the application of proteomic techniques in *S*. *paramamosain*, but also pave the way for the application of the third-generation sequencing technology in other crustacean.

## Results

### The quality examination of pooled RNA used for library construction and the evaluation of sequencing result

**Figure 1:**
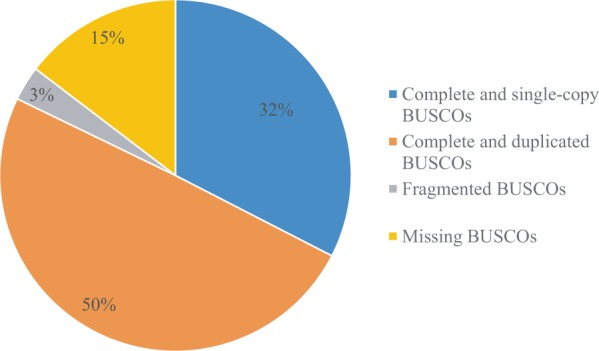
The evaluation of sequencing result analyzed by BUSCO software.

The quality of pooled total RNA extracted from twelve tissues was examined before library construction. The examined result indicated that the RNA was high quality and was appropriate for following experiment. The evaluation of sequencing result was carried out using 3 methods and the results were as follows: (1) The analysis result of BUSCO software revealed that 876 (82.2%) complete single-copy and duplicated BUSCOs, 34 (3.2%) fragmented BUSCOs (Benchmarking Universal Single Copy Orthologs), 156 (14.6%) missing BUSCOs (Fig. 1) (2) the aligned ratio of published transcriptome data sequenced by second-generation technology with that sequenced by Pacbio technology in this study was more than 77% (3) the sequences of published genes (*relish*, *dorsal*, *TGF-beta type I receptor* and *amine oxidase*) were consistent with sequencing result performed by Pacbio technology.

### Functional annotation of transcripts

**Figure 2:**
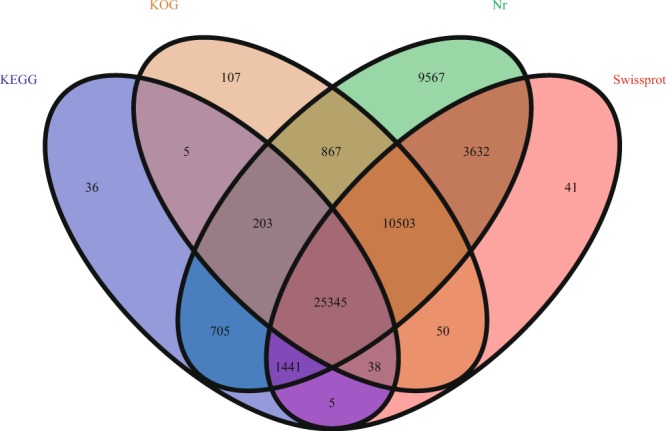
The venn diagram of annotated result in 4 different databases.

**Figure 3:**
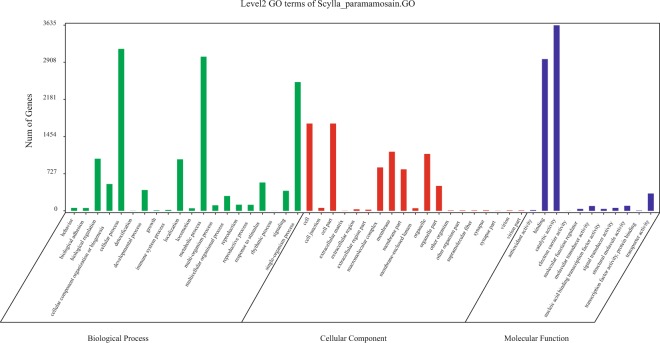
The GO annotation result.

The identified transcripts were blasted against protein database (Nr, Swiss-prot, KOG, and KEGG) and the result indicated that a total of 52,544 transcripts (66.5%) were annotated. Of which 52,262 transcripts were annotated in Nr database, 41,054 transcripts in Swiss-prot database, 37,117 transcripts in KOG database, and 27,777 transcripts in KEGG database. The venn diagram was shown in Fig. 2. GO analysis result indicated that 13,441 transcripts were annotated in biological process, 7,288 transcripts in molecular function, and 8,055 transcripts in cellular component. The detail information of GO annotation was shown in Fig. 3.

**Figure 4:**
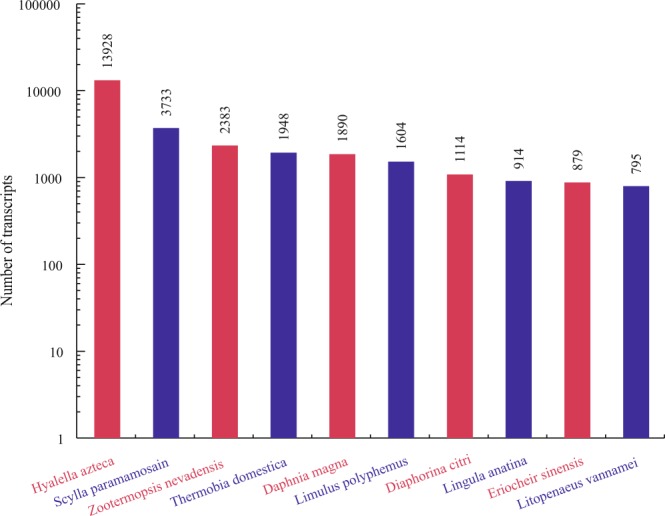
The top 10 species distribution of BLASTX results.

According to the annotated results, the species distribution of transcripts BLASTx matches against the Nr protein database was performed and the result indicated that the top 10 species all belong to invertebrate, which included *Hyalella Azteca*, *S*. *paramamosain*, *Zootermopsis nevadensis*, *Thermobia domestica*, *Daphnia magna*, *Limulus Polyphemus*, *Diaphorina citri*, *Lingula anatine*, *E*. *sinensis*, and *L*. *vannamei*. The detailed information of species distribution was shown in Fig. 4.

### Identification of long non-coding RNAs (lncRNAs)

**Figure 5:**
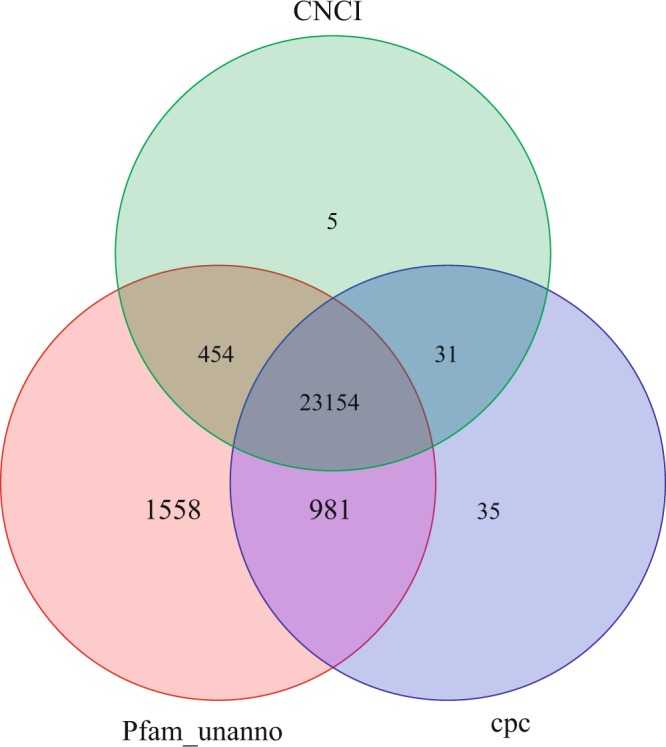
The venn diagram of LncRNAs prediction result by three softwares.

In this study, the coding potential of the unannotated transcripts was analyzed with three different bioinformatics softwares, Coding Potential Calculator (CPC), Coding-Non-Coding Index (CNCI), and Protein family (Pfam). The predicted result revealed that 24,201 LncRNAs were identified with the software of CPC, 23,644 LncRNAs with the software of CNCI and 26,147 LncRNAs with the software of Pfam, among which 23,154 common LncRNAs were predicted by three different bioinformatics software (Fig. 5).

### Identification of simple sequence repeats (SSRs)

**Figure 6:**
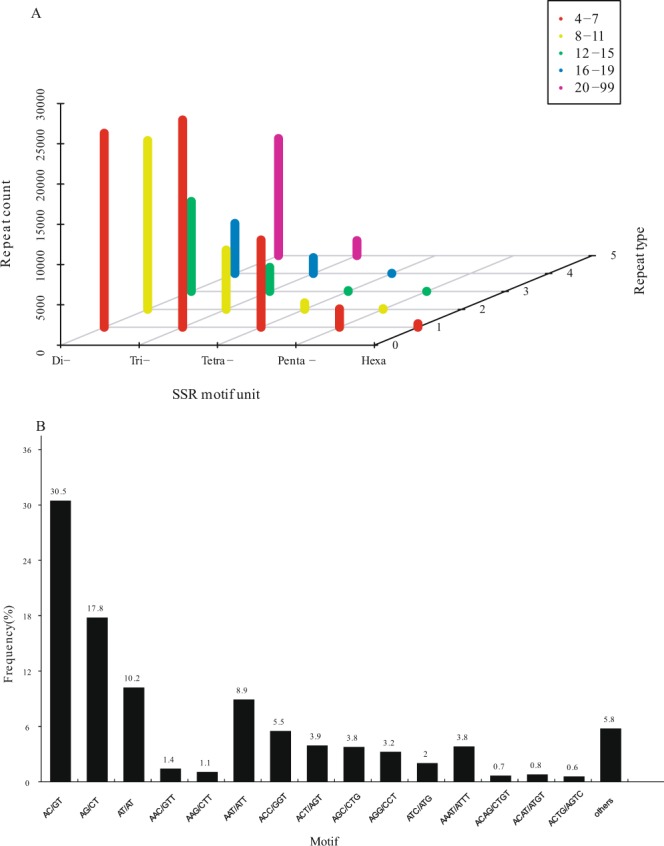
Distribution of simple sequence repeat (SSR) nucleotide classes among different nucleotide types found in the transcriptome of *S*. *paramamosain*.

A total of 131,561 SSRs were identified across all the transcripts, with 28,267 transcripts containing more than one SSR. Most of the SSRs identified were di-nucleotide repeats (58.53%), followed by the tri-nucleotide repeats (30.35%), tetra-nucleotide repeats (8.96%), penta-nucleotide repeats (1.82%) and Hexa-nucleotide (0.34%). In the di-nucleotide repeats, tri-nucleotide repeats, tetra-nucleotide repeats, the motif of AC/GT, AAT/ATT and AAAT/ATTT was the most dominant style, respectively. The detailed information was shown in Fig. 6A,B.

### The analysis of alternative splicing in transcriptome

**Figure 7:**
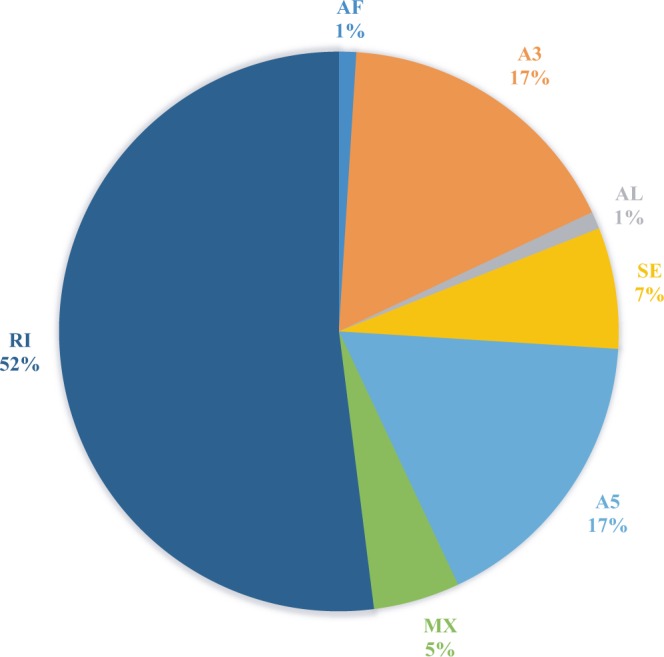
The statistics of alternative splicing events in the transcriptome of *S*. *paramamosain* shown in pie chart. Note: A3 represents alternative 3′ splice site, A5 represents alternative 5′ splice site, AF represents alternative first exon, AL represents alternative last exon, MX represents mutually exclusive exon, RI represents retained intron, SE represents skipping exon.

**Figure 8:**
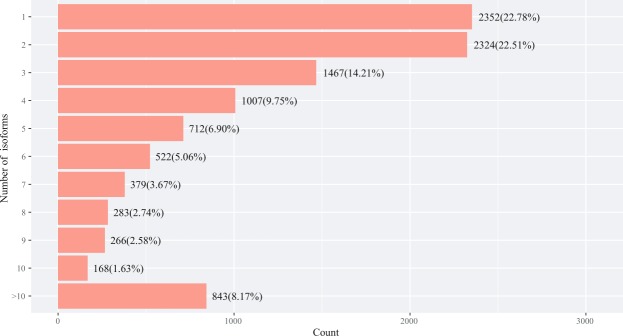
The statistics result of isoform of some genes.

**Figure 9:**
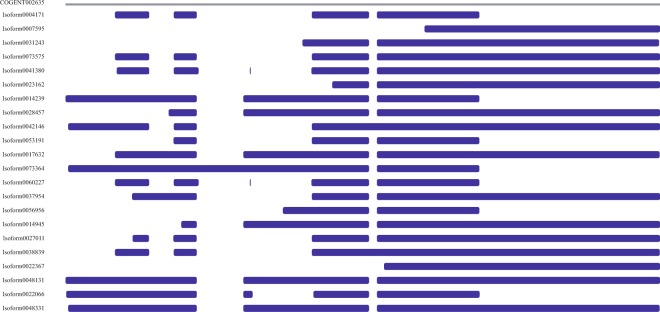
The sequence analysis of different isoforms of LIM domain-binding protein 3. Note: COGENT002635 represents the super-transcripts constructed with different isoforms of LIM domain-binding protein 3, the others represent the different isoforms.

**Figure 10:**
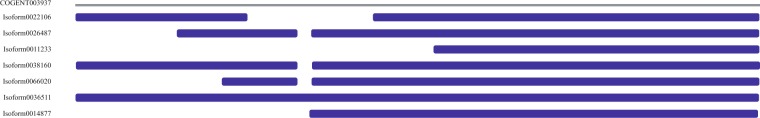
The sequence analysis of different isoforms of ferritin. Note: COGENT003937 represents the super-transcripts constructed with different isoforms of ferritin, the others represent the different isoforms.

The analysis result of alternative splicing indicated that there were seven different types existing in transcriptome, including 247 skipping exon (SE), 580 alternative 5′ splice site (A5), 600 alternative 3′ splice site (A3), 160 mutually exclusive exon (MX), 1780 retained intron (RI), 38 alternative first exon (AF), and 40 alternative last exon (AL), among which retained intron was the main type of alternative splicing, accounting for more than 5% (Fig. 7). The isoform analysis result indicated that the isoform number of some genes was more than ten (Fig. 8). For example, a total of 22 different isoforms of LIM domain-binding protein 3 were identified in this study and the sequence analysis result was shown in Fig. 9 (an example of RI). Additionally, 7 different isoforms of ferritin were identified and the sequence analysis result was shown in Fig. 10 (an example of A5).

### The validation of sequencing result with several published full-length genes

In order to validate the accuracy of sequencing result, several published genes, for example, *relish* (GI number MH047674.1), *dorsal* (GI number MH047675.1), *TGF-beta type I receptor* (GI number MH187960.1), and *amine oxidase* (GI number MG878093.1) were blasted against sequencing result using the blast software and the results indicated that the sequences of several published full-length genes were completely identical to the sequencing result except *dorsal* gene, which indicated the accuracy of sequencing result. The detailed blast results were shown in Supplemental File.

## Discussion

The obtainment of full-length gene is the first step to study gene function, but it can’t obtain on a large scale and is time consuming, labor intensive and expensive through rapid amplification of cDNA ends (RACE) technology in general. With the development of technology, the second-generation sequencing technologies are developed such as Illumine, Roche 454, Solexa, SOLID, the sequencing reads length of which is usually short. Though, part of full-length transcripts could be obtained through the transcriptome data sequenced by second-generation sequencing technologies on a large scale, majority of assembled transcripts is short and is not full-length. The third-generation sequencing technology is the most advanced technology, which could obtain full-length transcripts on a large scale. In this study, a total of 79005 high-quality unique transcripts is obtained, among which 50% transcripts is full-length, which is more efficient than RACE and the second-generation sequencing technology^30,32,33,48^. These full-length transcripts identified in this study will facilitate further study of *S*. *paramamosain*.

It is well known that the sequencing length of the third-generation sequencing technology could reach  as long as 2 Mb, avoiding the influence of the complex repeat motif. In this study, the longest transcript is 14701 bp and the N50 (an important parameter used for evaluating the quality of assembly) is 3160 bp, which is longer than that in *S*. *paramamosain* studies that used the second-generation sequencing technologies. For instance, in the gonad transcriptome, gill transcriptome, and hemocyte transcriptome of *S*. *paramamosain*, the N50 of assembled unigenes is only 477 bp, 1601bp, and 1488 bp, respectively^30–32^, which is far shorter than that in this study and indicates that the result of the third-generation sequencing technology is better than that of the second-generation sequencing technology.

Alternative splicing is an important way of regulating gene expression and plays vital roles in a variety of biological processes including sex differentiation and immunological resistance. In the study of *E*. *sinensis*, the two splice isoforms of the gene *fruitless* are obtained and could play important roles in sex-specific character development^66^. In the study of *L*. *vannamei*, a total of 6 *sex-lethal* splice isoforms are cloned used RACE technology and the different isoform may play different roles during embryo development^67^. In the study of *S*. *paramamosain*, the gene of *down syndrome cell adhesion molecule* (*Dscam*) is cloned and the bioinformatics result reveals  that it could encode as high as 36,736 unique isoforms to bind different pathogen to protect the crab from the pathogen infection^68^. However, in crustacean, the identification of alternative splicing on a large scale is scare because of the absence of genome information which makes the study of alternative splicing in crustacean difficult. Because of the longer sequencing length, the third-generation sequencing technology could obtain the full-length of transcripts, which provides the basis for the research of alternative splicing in *S*. *paramamosain*. In this study, the constructed sequencing library was consistent of 12 different tissues, therefore, more isoforms were identified comparing to the result that obtained using single tissue constructed sequencing library, which also indicated that different isoforms may play different roles in different tissues and the function of these isoforms needed further research. For example, a total of 6 different ferminazation-1 transcripts were identified in this study and their predicted protein sequences were completely identical to the protein sequences obtained through gonad transcriptome data in our laboratory (unpublished data). However, only 3 different ferminazation-1 transcripts (*fem-1a*, *fem-1b*, *fem-1c*) were identified in *E*. *sinensis* transcriptome data sequencing using second-generation sequencing technology, which indicated the third-generation sequencing technology is more efficient than second-generation sequencing technology in identifying isoforms.

It has been reported that the transcripts sequenced using the third-generation sequencing technology has more annotation rate than the second-generation sequencing technology in *L*.*vannamei*^48^. In published articles about *S*. *paramamosain* transcriptome, the annotation rate of transcripts was 59%, 15.7% and 48.38%, respectively^30–32^. In this study, the annotation rate of obtained transcripts was 66.5%, which was higher than that previously obtained using the second-generation sequencing technology and consistent with the result in *L*. *vannamei*^48^.

Previous studies have shown that raw data error rate of the third-generation sequencing technology is relatively high, but the raw data error rate could be corrected by the data of second-generation sequencing technology^69^. In this study, the raw data has been corrected by the transcriptome data sequenced using Illumina platform in our laboratory (unpublished result), which ensure the reality of the sequencing result. The consistent blast result of several published genes, *relish*, *dorsal*, *TGF-beta type I receptor*, *amine oxidase* with sequencing result also indicate the reliability of sequencing result in this study.

LncRNAs are non-coding RNAs that are longer than 200 nucleotides long and play vital roles in many physiological processes^70^. However, the identification of LncRNAs in *S*. *paramamosain* using the third-generation sequencing technology has never been reported. In this study, a total of 23154 common LncRNAs predicted by three softwares are obtained, which will facilitate the function study of these LncRNAs in *S*. *paramamosain*. In spite of the identification of LncRNAs through the third-generation sequencing technology in this study, the classification and false rate of identified LncRNAs could not be done because of the absence of genome data of *S*. *paramamosain*.

## Materials and Methods

### Samples

Healthy sexually adult male (n = 4) and female (n = 4) *S*. *paramamosain* (weight = 250 ± 10 g) were purchased from a local agricultural market in Xiamen, China. A total of 12 different tissues (gill, hepatopancreas, muscle, cerebral ganglion, eyestalk, thoracic ganglia, intestine, heart, testis, ovary, sperm reservoir and hemocyte) were collected. The total RNA was extracted using the E.Z.N.A.®. Total RNA Kit II (Omega, Norcross, GA, USA) following the protocol provided by the manufacturer. The integrity of the RNA was determined with the Agilent 2100 Bioanalyzer and agarose gel electrophoresis. The purity and concentration of the RNA were determined with the Nanodrop micro-spectrophotometer (Thermo Fisher, USA).

### SMRT library construction, sequencing, and quality control

mRNA was enriched by Oligo (dT) magnetic beads. Then the enriched mRNA was reverse transcribed into cDNA using Clontech SMARTer PCR cDNA Synthesis Kit (Takara, Shiga, Japan). PCR cycle optimization was used to determine the optimal amplification cycle number for the downstream large-scale PCR reactions. Then the optimized cycle number was used to generate double-stranded cDNA, followed by size selection using the Blue Pippin TM Size-Selection System to generate three libraries (1–2 kb, 2–3 kb, 3–6 kb). Then large-scale PCR was performed for the different size libraries for the next SMRT bell library construction. Different input amount of cDNA of size-selected samples was used to DNA damage repaired, end repaired, and ligated to sequencing adapters. The SMRT bell template was annealed to sequencing primer and bound to polymerase, and sequenced on the PacBio sequel platform by Gene Denovo Biotechnology Company (Guangzhou, China).

### Data processing

The raw sequencing reads of cDNA libraries were classified and clustered into transcript consensus using the SMRT Link v5.0.1 pipeline^71^ supported by Pacific Biosciences. Briefly, CCS (circular consensus sequence) reads were extracted out of subreads BAM file. Then CCS reads were classified into full-length non-chimeric (FL), non-full-length (nFL), chimeras, and short reads based on cDNA primers and polyA tail signal. Short reads were discarded. Subsequently, the full-length non-chimeric (FLNC) reads were clustered by Iterative Clustering for Error Correction (ICE) software to generate the cluster consensus isoforms. Then non full-length reads were used to polish the above obtained cluster consensus isoforms by Quiver software to finally obtain the FL polished high quality consensus sequences (accuracy ≥ 99%). The final transcriptome isoform sequences were filtered by removing the redundant sequences with software CD-HIT-v4.6.7 using a threshold of 0.99 identities.

### The evaluation of sequencing result and functional annotation of transcripts

The evaluation of sequencing result was performed through 3 different methods: (1) The protein sequences predicted from the sequencing result were analyzed by BUSCO v2.0 using arthropoda database to evaluate the completeness of sequencing result. (2) The published transcriptome data (SRR8792478, SRR8792479, SRR5814909, SRR5814910, SRR5814911, SRR5814912, SRR5814913, SRR5814914, SRR5814915, SRR5814916, SRR5814917) downloaded from NCBI database and the transcriptome results sequenced by our laboratory were aligned to sequencing result with bowtie2 software to evaluate the sequencing result. (3) Several recently published genes (*relish*: MH047674.1, *dorsal*: MH047675.1, *TGF-beta type I receptor*: MH187960.1 and *amine oxidase*: MG878093.1) were compared with the sequencing result to validate the accuracy of sequencing result. Basic annotation of transcripts includes protein functional annotation, pathway annotation, COG/KOG functional annotation and Gene Ontology (GO) annotation. To annotate the transcripts, transcripts were blasted against the NCBI non-redundant protein (Nr) database (http://www.ncbi.nlm.nih.gov), the Swiss-Prot protein database (http://www.expasy.ch/sprot), the Kyoto Encyclopedia of Genes and Genomes (KEGG) database (http://www.genome.jp/kegg), and the COG/KOG database (http://www.ncbi.nlm.nih.gov/COG) with BLASTx program (http://www.ncbi.nlm.nih.gov/BLAST/) at an E-value threshold of 1e–5 to evaluate sequence similarity with genes of other species. GO annotation was analyzed by Blast2GO software^72^ with Nr annotation results of transcripts. Transcripts ranking the first 20 highest score and no shorter than 33 HSPs (High-scoring Segment Pair) hits were selected to conduct Blast2GO analysis. Then, functional classification of transcripts was performed using WEGO software^73^.

### Characterization of long non-coding RNAs

CNCI v2.0^74^, pfam^75^ and CPC v1.0^76^ were used to assess the protein-coding potential of transcripts without annotations by default parameters for potential long non-coding RNAs. To better annotate lncRNAs in evolution level, the software Infernal (http://eddylab.org/infernal/) was used in sequence alignment. The lncRNAs were classified by secondary structures and sequence conservation.

### Alternative splicing detection

To analyze alternative splicing events of transcript isoforms, COding GENome reconstruction Tool (Cogent) was firstly used to partition transcripts into gene families based on k-mer similarity and reconstructed each family into a coding reference genome based on De Bruijn graph methods. Then SUPPA tool was used to analyze alternative splicing events of transcript isoforms.

### Identification of SSRs

**Figure 11:**
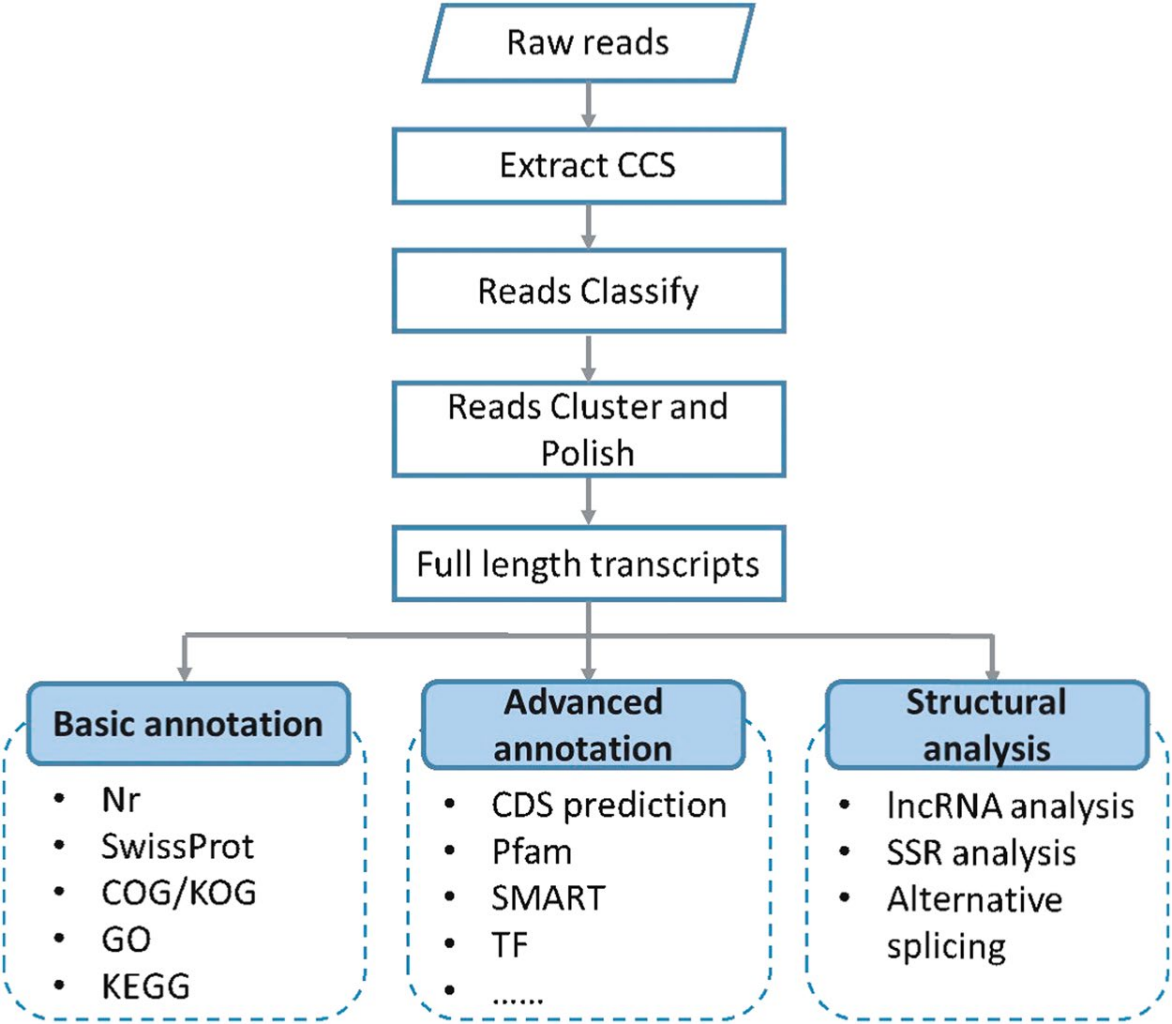
The overall analysis pipeline performed in this study.

The SSR identification was analyzed employing the software of MISA v1.0 (http://pgrc.ipk-gatersleben.de/misa/) 64 with default parameters in the whole transcriptome. The primers used for PCR were designed using primer3 with default parameters. The overall analysis pipeline was shown in Fig. 11.

## Supplementary information

The analysis result of published relish, dorsal, and TGF-beta type I receptor genes with sequencing result

## References

[1] Wei Banghong, Yang Zhigang, Wang Jianyi, Chen Aqin, Shi Qiuyan, Cheng Yongxu (2017). Effects of dietary lipids on the hepatopancreas transcriptome of Chinese mitten crab (Eriocheir sinensis). PLOS ONE.

[2] Wang Y (2017). Integrated analysis of mRNA-seq in the haemocytes of *Eriocheir sinensis* in response to *Spiroplasma eriocheiris* infection. Fish Shellfish Immunol.

[3] Zhang D (2018). Comparative transcriptome analysis of *Eriocheir japonica sinensis* response to environmental salinity. PloS One.

[4] Yu N (2018). Growth, energy metabolism and transcriptomic responses in Chinese mitten crab (*Eriocheir sinensis*) to benzo[alpha]pyrene (BaP) toxicity. Aquat Toxicol.

[5] Zhang C (2018). Hemolymph transcriptome analysis of Chinese mitten crab (*Eriocheir sinensis*) with intact, left cheliped autotomy and bilateral eyestalk ablation. Fish Shellfish Immunol.

[6] Jia ZH (2018). Transcriptome sequencing reveals the involvement of reactive oxygen species in the hematopoiesis from Chinese mitten crab *Eriocheir sinensis*. Dev Comp Immunol.

[7] Fu C (2017). Comparative transcriptome analysis reveals related regulatory mechanisms of androgenic gland in *Eriocheir sinensis*. Biomed Res Int.

[8] Li Gen-Liang, Qian Hui (2017). Transcriptome using Illumina sequencing reveals the traits of spermatogenesis and developing testes in Eriocheir sinensis. PLOS ONE.

[9] Chen XW, Wang J, Yue WC, Liu JS, Wang CH (2017). Hepatopancreas transcriptome analysis of Chinese mitten crab (*Eriocheir sinensis*) with white hepatopancreas syndrome. Fish Shellfish Immunol.

[10] Hui M, Cui ZX, Liu Y, Song CW (2017). Transcriptome profiles of embryos before and after cleavage in *Eriocheir sinensis*: identification of developmental genes at the earliest stages. Chin J Oceanol Limn.

[11] Xu Y (2017). Comparative transcriptome sequencing of the hepatopancreas reveals differentially expressed genes in the precocious juvenile Chinese mitten crab, *Eriocheir sinensis* (Crustacea: Decapoda). Aquaculture Res.

[12] Du FK, Li Y, Wen ZX, Chen RG, Xu P (2017). Development of simple sequence repeats (SSR) by transcriptome in Chinese mitten crab (*Eriocheir sinensis* H. Milne Edwards). Pak J Zool.

[13] Zhu FJ, Hu K, Yang ZY, Yang XL (2017). Comparative transcriptome analysis of the hepatopancreas of *Eriocheir sinensis* following oral gavage with enrofloxacin. Can J Fish Aquat Sci.

[14] Hui Min, Liu Yuan, Song Chengwen, Li Yingdong, Shi Guohui, Cui Zhaoxia (2014). Transcriptome Changes in Eriocheir sinensis Megalopae after Desalination Provide Insights into Osmoregulation and Stress Adaption in Larvae. PLoS ONE.

[15] Song Ya-Nan, Shi Li-Li, Liu Zhi-Qiang, Qiu Gao-Feng (2014). Global analysis of the ovarian microRNA transcriptome: implication for miR-2 and miR-133 regulation of oocyte meiosis in the Chinese mitten crab, Eriocheir sinensis (Crustacea:Decapoda). BMC Genomics.

[16] Sun Yan, Zhang Yichen, Liu Yichen, Xue Shuxia, Geng Xuyun, Hao Tong, Sun Jinsheng (2014). Changes in the Organics Metabolism in the Hepatopancreas Induced by Eyestalk Ablation of the Chinese Mitten Crab Eriocheir sinensis Determined via Transcriptome and DGE Analysis. PLoS ONE.

[17] Li EC (2014). Transcriptome sequencing revealed the genes and pathways involved in salinity stress of Chinese mitten crab, *Eriocheir sinensis*. Physiol Genomics.

[18] Cui, Z. X. *et al*. Transcriptome profiling analysis on whole bodies of microbial challenged *Eriocheir sinensis* larvae for immune gene identification and SNP development. *PloS One* **8**, doi:1371/journal.pone.0082156 (2013). 10.1371/journal.pone.0082156 PMC3852986 24324760

[19] He Lin, Jiang Hui, Cao Dandan, Liu Lihua, Hu Songnian, Wang Qun (2013). Comparative Transcriptome Analysis of the Accessory Sex Gland and Testis from the Chinese Mitten Crab (Eriocheir sinensis). PLoS ONE.

[20] He Lin, Wang Yuan-Li, Li Qing, Yang Hong-Dan, Duan Ze-Lin, Wang Qun (2015). Profiling microRNAs in the testis during sexual maturation stages in Eriocheir sinensis. Animal Reproduction Science.

[21] Ou JT (2012). Identification and comparative analysis of the *Eriocheir sinensis* microRNA transcriptome response to *Spiroplasma eriocheiris* infection using a deep sequencing approach. Fish Shellfish Immunol.

[22] Liu L (2018). Transcriptomic analysis of *Portunus trituberculatus* reveals a critical role for WNT4 and WNT signalling in limb regeneration. Gene.

[23] Wang Z (2018). *De novo* transcriptome sequencing and analysis of male and female swimming crab (*Portunus trituberculatus*) reproductive systems during mating embrace (stage II). BMC Genet.

[24] Li Y (2017). RNA-Seq analysis of the antioxidant status and immune response of *Portunus trituberculatus* following aerial exposure. Mar Biotechnol (NY).

[25] Meng XL, Liu P, Jia FL, Li J, Gao BQ (2015). *De novo* transcriptome analysis of *Portunus trituberculatus* ovary and testis by RNA-Seq: identification of genes involved in gonadal development. PloS One.

[26] Yang Y (2015). Ovarian transcriptome analysis of *Portunus trituberculatus* provides insights into genes expressed during phase III and IV development. PloS One.

[27] Lv J (2014). Transcriptome analysis of the *Portunus trituberculatus*: *de novo* assembly, growth-related gene identification and marker discovery. PloS One.

[28] Wang W, Wu X, Liu Z, Zheng H, Cheng Y (2014). Insights into hepatopancreatic functions for nutrition metabolism and ovarian development in the crab *Portunus trituberculatus*: gene discovery in the comparative transcriptome of different hepatopancreas stages. PloS One.

[29] Lv J (2013). Transcriptome analysis of *Portunus trituberculatus* in response to salinity stress provides insights into the molecular basis of osmoregulation. PloS One.

[30] Yang X (2018). Comparative transcriptome analysis provides insights into differentially expressed genes and long non-coding RNAs between ovary and testis of the mud crab (*Scylla paramamosain*). Mar Biotechnol (NY).

[31] Zhu F, Qian X, Ma X (2018). Comparative transcriptomic analysis of crab hemocytes in response to white spot syndrome virus or *Vibrio alginolyticus* infection. Fish Shellfish Immunol.

[32] Liu S (2017). Transcriptome analysis of mud crab (*Scylla paramamosain*) gills in response to mud crab reovirus (MCRV). Fish Shellfish Immunol.

[33] Jiang Q (2017). Transcriptome profiling of claw muscle of the mud crab (*Scylla paramamosain*) at different fattening stages. PloS One.

[34] Waiho K (2017). Transcriptome analysis and differential gene expression on the testis of orange mud crab, *Scylla olivacea*, during sexual maturation. PloS One.

[35] Alexander J, Oliphant A, Wilcockson DC, Webster SG (2018). Functional identification and characterization of the diuretic hormone 31 (DH31) signaling system in the green shore crab, *Carcinus maenas*. Front Neurosci.

[36] Verbruggen B (2015). *De novo* assembly of the *Carcinus maenas* transcriptome and characterization of innate immune system pathways. BMC Genomics.

[37] Shyamal S, Das S, Guruacharya A, Mykles DL, Durica DS (2018). Transcriptomic analysis of crustacean molting gland (Y-organ) regulation via the mTOR signaling pathway. Sci Rep.

[38] Das S, Vraspir L, Zhou W, Durica DS, Mykles DL (2018). Transcriptomic analysis of differentially expressed genes in the molting gland (Y-organ) of the blackback land crab, *Gecarcinus lateralis*, during molt-cycle stage transitions. Comp Biochem Physiol Part D Genomics Proteomics.

[39] Martin LA, Das S, Mykles DL (2016). *De novo* transcriptome assembly and analysis of the molting gland in blackback land crab, *Gecarcinus lateralis*, throughout various molt stages. Integr Comp Biol.

[40] Das S, Pitts NL, Mudron MR, Durica DS, Mykles DL (2016). Transcriptome analysis of the molting gland (Y-organ) from the blackback land crab, *Gecarcinus lateralis*. Comp Biochem Phys D.

[41] Mykles DL, Pitts NL, Das S, Durica DS (2015). Transcriptome analyses of intermolt and premolt molting glands from the blackback land crab, *Gecarcinus lateralis*. Integr Comp Biol.

[42] Zhang Y (2018). Transcriptome sequencing and molecular markers discovery in the gonads of *Portunus sanguinolentus*. Sci Data.

[43] Zhang Y (2018). Transcriptome-seq provides insights into sex-preference pattern of gene expression between testis and ovary of the crucifix crab (*Charybdis feriatus*). Physiol Genomics.

[44] Zheng Z (2018). Comparative transcriptomic analysis of shrimp hemocytes in response to acute hepatopancreas necrosis disease (AHPND) causing *Vibrio parahemolyticus* infection. Fish Shellfish Immunol.

[45] Maralit BA, Jaree P, Boonchuen P, Tassanakajon A, Somboonwiwat K (2018). Differentially expressed genes in hemocytes of *Litopenaeus vannamei* challenged with *Vibrio parahaemolyticus* AHPND (VPAHPND) and VPAHPND toxin. Fish Shellfish Immunol.

[46] Lu X (2018). Identification of SNP markers associated with tolerance to ammonia toxicity by selective genotyping from *de novo* assembled transcriptome in *Litopenaeus vannamei*. Fish Shellfish Immunol.

[47] Qin Z (2018). Transcriptome analysis of Pacific white shrimp (*Litopenaeus vannamei*) challenged by *Vibrio parahaemolyticus* reveals unique immune-related genes. Fish Shellfish Immunology.

[48] Zeng D (2018). Single-molecule long-read sequencing facilitates shrimp transcriptome research. Sci Rep.

[49] Ding Z, Jin M, Ren Q (2018). Transcriptome analysis of *Macrobrachium rosenbergii* intestines under the white spot syndrome virus and poly (I:C) challenges. PloS One.

[50] Cao J (2017). Transcriptome profiling of the *Macrobrachium rosenbergii* lymphoid organ under the white spot syndrome virus challenge. Fish Shellfish Immunol.

[51] Rao R (2016). A transcriptome study on *Macrobrachium rosenbergii* hepatopancreas experimentally challenged with white spot syndrome virus (WSSV). J Invertebr Pathol.

[52] Nguyen Thanh H, Zhao L, Liu Q (2014). *De novo* transcriptome sequencing analysis and comparison of differentially expressed genes (DEGs) in *Macrobrachium rosenbergii* in China. PloS One.

[53] Zhao C (2018). A transcriptome study on *Macrobrachium nipponense* hepatopancreas experimentally challenged with white spot syndrome virus (WSSV). PloS One.

[54] Jin S (2013). Transcriptome analysis of androgenic gland for discovery of novel genes from the oriental river prawn, *Macrobrachium nipponense*, using Illumina Hiseq 2000. PloS One.

[55] Wang J (2018). Identification of novel EST-SSR markers by transcriptome sequencing in ridgetail white prawn *Exopalaemon carinicauda*. Genes Genomics.

[56] Ge Q (2017). Transcriptome analysis of the hepatopancreas in *Exopalaemon carinicauda* infected with an AHPND-causing strain of *Vibrio parahaemolyticus*. Fish Shellfish Immunol.

[57] Li J, Li J, Chen P, Liu P, He Y (2015). Transcriptome analysis of eyestalk and hemocytes in the ridgetail white prawn *Exopalaemon carinicauda*: assembly, annotation and marker discovery. Mol Biol Rep.

[58] Lou F, Gao T, Cai S, Han Z (2018). *De novo* assembly and annotation of the whole transcriptome of *Oratosquilla oratoria*. Mar Genomics.

[59] Yan H (2018). *De novo* transcriptome analysis and differentially expressed genes in the ovary and testis of the Japanese mantis shrimp *Oratosquilla oratoria* by RNA-Seq. Comp Biochem Physiol Part D Genomics Proteomics.

[60] Zhang D (2018). Transcriptome analysis of hepatopancreas from the Cr (VI)-stimulated mantis shrimp (*Oratosquilla oratoria*) by Illumina paired-end sequencing: assembly, annotation, and expression analysis. J Agric Food Chem.

[61] McGrath, L. L., Vollmer, S. V., Kaluziak, S. T. & Ayers, J. *De novo* transcriptome assembly for the lobster *Homarus americanus* and characterization of differential gene expression across nervous system tissues. *BMC Genomics* **17**, 10.1186/s12864-016-2373-3 (2016). 10.1186/s12864-016-2373-3 PMC4715275 26772543

[62] Haas BJ (2013). *De novo* transcript sequence reconstruction from RNA-seq using the Trinity platform for reference generation and analysis. Nat Protoc.

[63] Abdelrahman H (2017). Aquaculture genomics, genetics and breeding in the United States: current status, challenges, and priorities for future research. BMC Genomics.

[64] Zhang Y (2018). Transcriptome analyses reveal *Litopenaeus vannamei* hemocytes response to lipopolysaccharide. Fish Shellfish Immunol.

[65] Roberts RJ, Carneiro MO, Schatz MC (2013). The advantages of SMRT sequencing. Genome Biol.

[66] Li P, Liu Y, Luo D, Song C, Cui Z (2017). Two spliced isoforms of the sex-determination gene *fruitless* in the Chinese mitten crab *Eriocheir sinensis*. Comp Biochem Physiol B Biochem Mol Biol.

[67] Lopez-Cuadros I (2018). Isolation of the sex-determining gene *Sex-lethal* (*Sxl*) in *Penaeus (Litopenaeus) vannamei* (Boone, 1931) and characterization of its embryogenic, gametogenic, and tissue-specific expression. Gene.

[68] Li W (2017). Characterize a typically dscam with alternative splicing in mud crab Scylla paramamosain. Fish Shellfish Immunol.

[69] Hackl T, Hedrich R, Schultz J, Forster F (2014). proovread: large-scale high-accuracy PacBio correction through iterative short read consensus. Bioinformatics.

[70] Kapranov P (2007). RNA maps reveal new RNA classes and a possible function for pervasive transcription. Science.

[71] Gordon SP (2015). Widespread polycistronic transcripts in fungi revealed by single-molecule mRNA sequencing. PloS One.

[72] Conesa A (2005). Blast2GO: a universal tool for annotation, visualization and analysis in functional genomics research. Bioinformatics.

[73] Ye J (2018). WEGO 2.0: a web tool for analyzing and plotting GO annotations, 2018 update. Nucleic Acids Res.

[74] Sun L (2013). Utilizing sequence intrinsic composition to classify protein-coding and long non-coding transcripts. Nucleic Acids Res.

[75] Sonnhammer EL, Eddy SR, Durbin R (1997). Pfam: a comprehensive database of protein domain families based on seed alignments. Proteins.

[76] Kong L (2007). CPC: assess the protein-coding potential of transcripts using sequence features and support vector machine. Nucleic Acids Res.

