# Supplementary material for: The Single-molecule long-read sequencing of Scylla paramamosain
Source: Sci Rep. 2019 Aug 27;9:12401. doi: 10.1038/s41598-019-48824-8 (PMC6711964; doi:10.1038/s41598-019-48824-8)
Supplement: Supplementary file 1 — The analysis result of published relish, dorsal, and TGF-beta type I receptor genes with sequencing result [file 41598_2019_48824_MOESM1_ESM.pdf]

The Single-molecule long-read sequencing of *Scylla paramamosain*

Haifu Wan[a], Xiwei Jia[a], Pengfei Zou[a], Ziping Zhang[b*], Yilei Wang[a*]

[a] Fisheries College, Jimei University, Xiamen, 361021, P. R. China

[b] College of Animal Science, Fujian Agriculture and Forestry University, Fuzhou, 350002, P. R. China

*Corresponding authors:

Prof. Yilei Wang, Email: ylwang@jmu.edu.cn

Prof. Ziping Zhang, E-mail: zhangziping@fafu.edu.cn

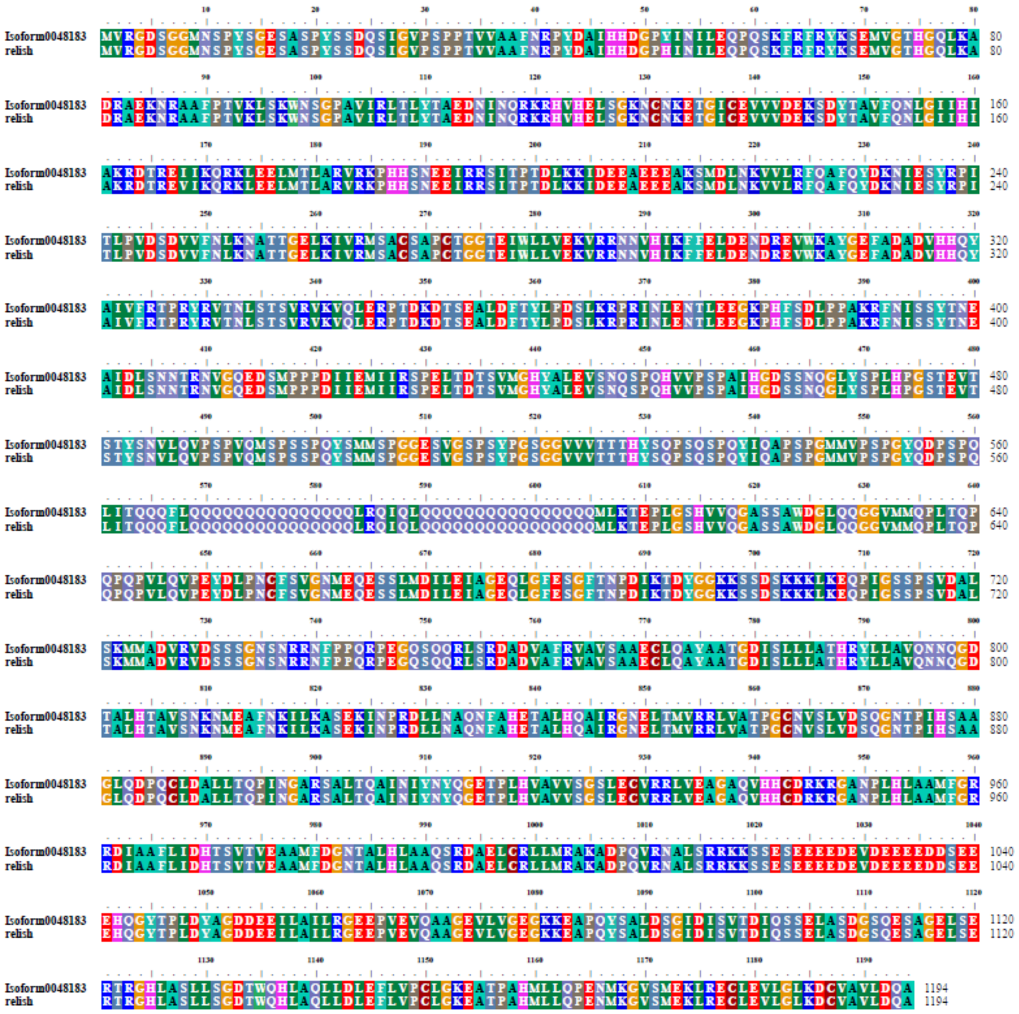

Supplement Figure1. The analysis result of published *relish* gene with sequencing result

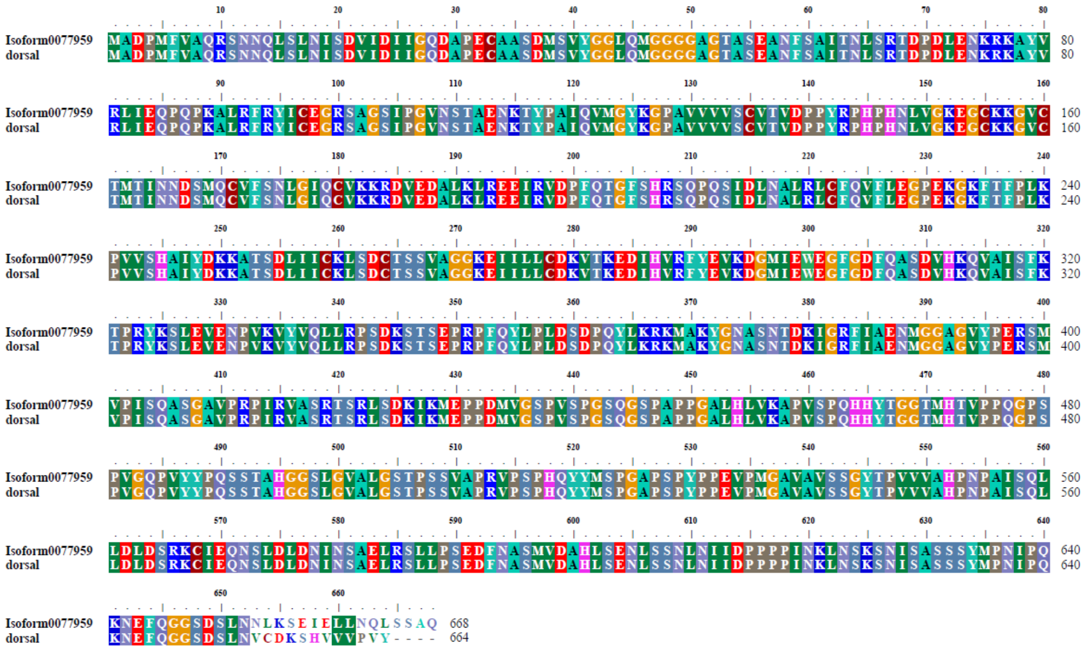

Supplement Figure 2. The analysis result of published *dorsal* gene with sequencing result

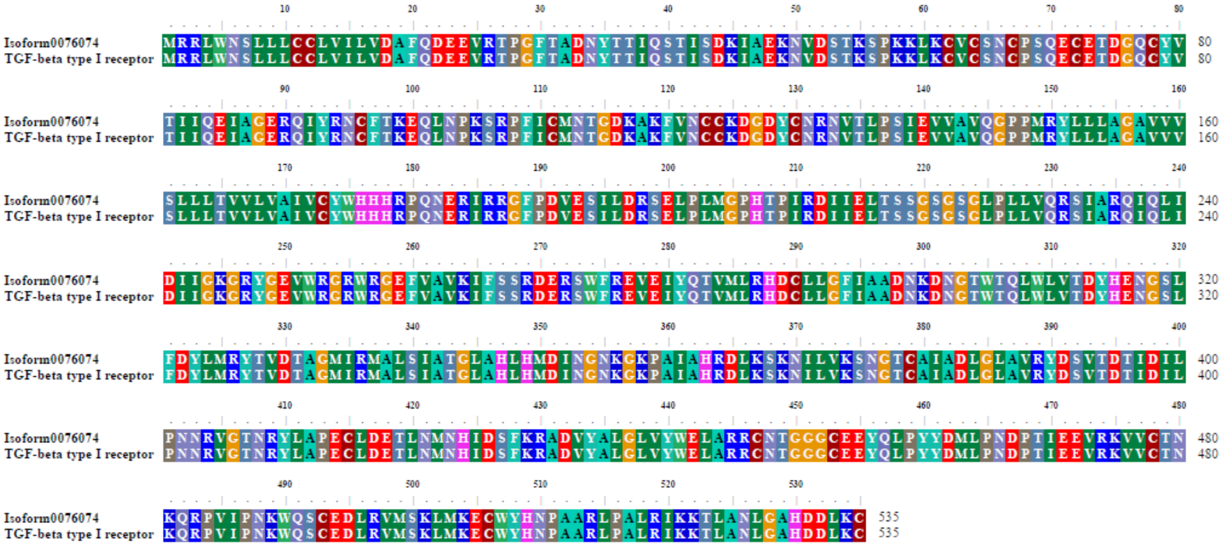

Supplement Figure 3. The analysis result of published *TGF-beta type I receptor* gene with sequencing result
